# Plasma Cabozantinib Level Measurement in Patients with Renal Cell and Hepatocellular Carcinomas Using a Simple HPLC–UV Method Suitable for Clinical Application

**DOI:** 10.3390/curroncol30050367

**Published:** 2023-05-08

**Authors:** Takeo Yasu, Yoshito Gando, Ryosuke Nishijima, Risa Ikuta, Motofumi Suzuki, Mikio Shirota

**Affiliations:** 1 Department of Medicinal Therapy Research, Pharmaceutical Education and Research Center, Meiji Pharmaceutical University, Tokyo 204-8588, Japan; 2 Bokutoh Hospital-Meiji Pharmaceutical University Joint Research Center, Tokyo 130-8575, Japan; 3 Department of Clinical Laboratory, Tokyo Metropolitan Bokutoh Hospital, Tokyo 130-8575, Japan; 4 Department of Urology, Tokyo Metropolitan Bokutoh Hospital, Tokyo 130-8575, Japan; 5 Department of Pharmacy, Tokyo Metropolitan Bokutoh Hospital, Tokyo 130-8575, Japan

**Keywords:** cabozantinib, high-performance liquid chromatography–ultraviolet, plasma concentration, therapeutic drug monitoring

## Abstract

Cabozantinib, which is used to treat renal cell and hepatocellular carcinomas, is often associated with dose-dependent adverse events. Monitoring the levels of cabozantinib in the blood may maximize the therapeutic effect and prevent serious adverse events. In this study, we developed a high-performance liquid chromatography–ultraviolet (HPLC–UV) method of measuring plasma cabozantinib concentration. Human plasma samples (50 µL) were processed by simple deproteinization with acetonitrile, followed by chromatographic separation on a reversed-phase column with an isocratic mobile phase of 0.5% KH₂PO_4_ (pH 4.5) and acetonitrile (43:57, *v*/*v*) at a flow rate of 1.0 mL/min, with a 250 nm ultraviolet detector. The calibration curve was linear over the concentration range (0.05–5 µg/mL) with a coefficient of determination of 0.99999. The accuracy of the assay ranged from −4.35% to 0.98%, and recovery was >96.04%. The measurement time was 9 min. These findings confirm the effectiveness of this HPLC–UV method for cabozantinib quantification in human plasma, which is sufficiently simple for use for monitoring patients in clinical settings.

## 1. Introduction

Cabozantinib is an oral vascular endothelial growth factor receptor (VEGFR) tyrosine kinase inhibitor that inhibits MET, VEGFR, AXL, and other tyrosine kinases. It has been approved for use in treating advanced renal cell carcinoma (RCC) and hepatocellular carcinoma (HCC) [1]. Cabozantinib is administered for advanced or metastatic RCC and unresectable HCC following the use of other systemic therapies, with a recommended initial dose of 60 mg. However, the incidence of cabozantinib dose reduction was 62% in both the METEOR [2] and CELESTIAL trials [3], due to several treatment-related adverse events (AEs). Furthermore, in a phase 2 trial conducted in Japan, 91% of patients with both RCC and HCC required cabozantinib dose reduction owing to AEs [4,5]. The risk of AEs depends on cabozantinib exposure [6]. Plasma concentrations of cabozantinib in Japanese patients have been shown to be 30% higher than those in non-Japanese patients [7]. Moreover, Japanese patients with HCC have been reported to have large individual differences in cabozantinib exposure [8]. One reason for the increased concentration of cabozantinib in the plasma of Japanese patients, compared with their European and American counterparts, is a difference in body size. Therefore, higher levels may also be observed in non-Japanese Asian populations. To ensure its effectiveness, the recommended cabozantinib trough plasma concentration is >0.53 µg/mL [9], but an upper limit of the target plasma concentration to avoid AEs has not been specified. Therapeutic drug monitoring (TDM)-based dose adjustment for personalized medicine is necessary for effective and safe cabozantinib therapy. Pharmacokinetic studies of cabozantinib in clinical TDM using liquid chromatography with tandem mass spectrometry (LC–MS/MS) has been used to measure cabozantinib concentrations in human plasma [10]. LC–MS/MS is an excellent instrument for rapid and sensitive quantification of drugs in a variety of biological matrices; however, the high cost of this instrument limits its use in general hospital settings. High-performance liquid chromatography–ultraviolet (HPLC–UV) is more affordable than LC–MS/MS and is more suitable for routine TDM. Recently, an HPLC–UV method was reported to measure plasma cabozantinib concentrations [11]. In this study, we developed a simple and sensitive HPLC–UV method to quantify the concentration of cabozantinib in human plasma for clinical application with a more convenient pretreatment than the previous HPLC–UV method [11]. To enable this assay to be applied in personalized medicine in a large number of patients, the effect of frequently used concomitant medications was assessed in patients receiving cabozantinib using real-world data.

## 2. Materials and Methods

### 2.1. Reagents and Chemicals

**Figure 1:**
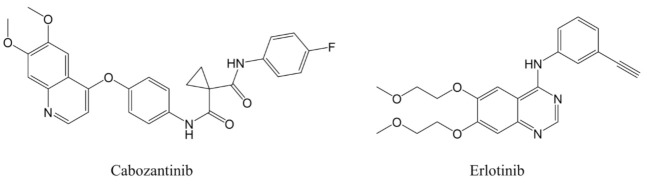
Chemical structures of cabozantinib (**left**) and erlotinib (**right**).

Cabozantinib and erlotinib (internal standard, IS) were obtained from Toronto Research Chemicals Inc. (Toronto, Ontario, Canada) and Tokyo Chemical Industry Co., Ltd. (Tokyo, Japan), respectively (Figure 1). The HPLC mobile phases were HPLC-grade acetonitrile, methanol, water (Kanto Chemical, Co., Inc., Tokyo, Japan), and KH₂PO₄ (Wako Pure Chemical Industries, Ltd., Osaka, Japan). Pooled human plasma collected with EDTA-2Na was purchased from Cosmo Bio Co., Ltd. (Tokyo, Japan).

### 2.2. Equipment and Chromatographic Conditions

The HPLC system consisted of a pump (PU-4180; Jasco, Tokyo, Japan), an ultraviolet detector (UV-4075; Jasco), and an autosampler (AS-4550; Jasco). The HPLC column was a Capcell Pak C18 MG II (Osaka Soda Co., Ltd., Tokyo, Japan) reversed-phase column (250 nm × 4.6 nm i.d.) and Capcell Pak C18 MG II guard column (10 mm × 4.0 mm; Osaka Soda) at a temperature of 20 °C. The mobile phase was 0.5% KH₂PO₄ (pH 4.5) and acetonitrile (43:57, *v*/*v*), and the flow rate was 1.0 mL/min. Sample detection was carried out at 250 nm.

### 2.3. Preparation of Stock and Working Solutions

Stock solutions of cabozantinib (0.5 mg/mL) and the IS (1 mg/mL) were prepared in acetonitrile and methanol, respectively. Working solutions of cabozantinib (0.25, 0.5, 2.5, 5, 12.5, 25 μg/mL) were prepared by diluting the stock solution of cabozantinib with acetonitrile. IS stock solution was diluted with methanol to make a working solution of IS with a concentration of 2.5 μg/mL. The stock and working solutions were stored at −60 °C in the dark.

### 2.4. Sample Preparation

**Figure 2:**
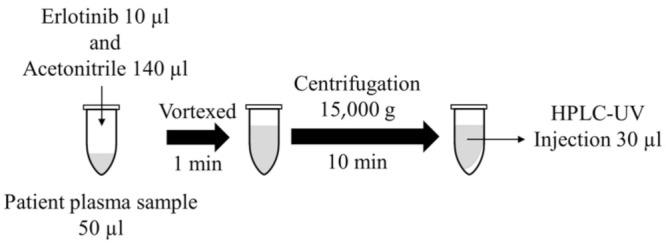
Sample preparation.

Before the analyses, the human plasma and working solutions were thawed and vortexed. The samples were deproteinized with acetonitrile. Blank plasma (50 µL) was spiked with 10 µL of cabozantinib. The spiked sample was vortexed for 15 s, followed by the addition of 10 µL IS and 130 µL acetonitrile, chilled to −20 °C, and vortexed for 1 min. The sample was centrifuged at 15,000 *g* for 10 min at 4 °C, and 30 µL of the supernatant was directly injected into the HPLC system for analysis (Figure 2).

### 2.5. Calibration and Validation

Accuracy and linearity were evaluated by analyzing a set of standard samples ranging from 0.05 to 5 µg/mL. The intra- and interday precision and accuracy were determined by repeated analysis of five sets of samples spiked with six concentrations of cabozantinib (0.05, 0.1, 0.5, 1, 2.5, and 5 µg/mL) within the same day or on five consecutive days, respectively. The recovery of the assay was determined for five sets of samples at each concentration, using six concentrations. The limit of quantification (LOQ) was defined as the lowest concentration on the calibration curve, and the limit of detection (LOD) was defined as the concentration that caused a signal-to-noise ratio ≥ 3 (S/N ≥ 3/1).

### 2.6. Stability

The stability of cabozantinib in human plasma was determined by analyzing the quality control (QC) samples at three different concentrations (0.05, 1, and 5 µg/mL) at various temperatures and times. The QC samples were analyzed against a calibration curve prepared using freshly spiked analytes, and the measured concentrations were compared to the nominal values. The QC samples were analyzed for benchtop stability (6 h at 20 °C), short-term stability (24 h at 4 °C), long-term stability (1 month at −60 °C), and three freeze–thaw cycles (freezing below −60 °C and thawing at room temperature). Five replicates were analyzed for each QC sample. For cabozantinib to be considered acceptably stable, its concentration had to be within ±15% of the freshly prepared standard.

### 2.7. Survey of Concomitant Medications in Patients Who Received Cabozantinib Using Real-World Data

We used the JMDC database, a nationwide hospital claims database in Japan. All patient data are encrypted before being added to the database. Between August 2021 and July 2022, data of 2,073,988 patients with malignant neoplasms were recorded in the database. Among these patients, only those who were prescribed cabozantinib were included in this analysis. The patient characteristics were based on the first prescription of cabozantinib. The extracted data included age, sex, type of malignant neoplasm, and concomitant medications used during the prescription of cabozantinib.

### 2.8. Selectivity

Based on real-world data, interference testing was conducted with frequent combination drugs, cabozantinib, and the IS. Interference was defined as a retention time within ±1 min of cabozantinib administration and IS retention time, as reported by Koeber et al. [12].

### 2.9. Clinical Application

After obtaining written informed consent from the patient receiving cabozantinib, we collected blood samples. Plasma and serum samples were obtained by centrifuging the blood samples at 3,000 *g* for 5.5 min, and the plasma and serum were stored at −80 °C until analysis. This study was approved by the institutional review board of Tokyo Metropolitan Bokutoh Hospital (#04–101) and was conducted in accordance with the Declaration of Helsinki.

## 3. Results

### 3.1. Chromatography

**Figure 3:**
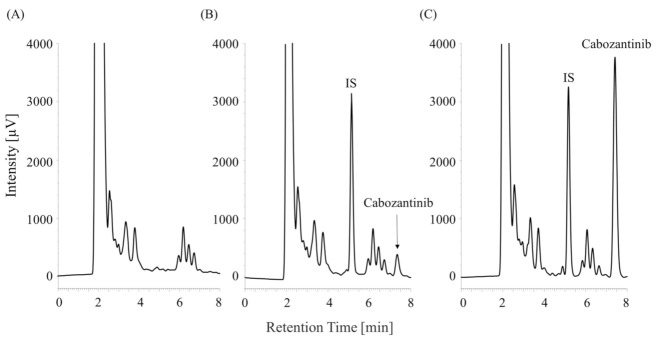
Chromatograms of the (**A**) blank plasma sample, (**B**) plasma sample containing 0.05 µg/mL of cabozantinib, and (**C**) plasma sample containing 0.5 µg/mL of cabozantinib.

Representative chromatograms of the blank human plasma samples are shown in Figure 3A. Cabozantinib and the IS were well separated from the coextracted materials under the chromatographic conditions in this study at retention times of 7.2 and 5.2 min, respectively. No interfering peaks from endogenous human plasma components were observed at the retention times of cabozantinib or the IS (Figure 3B,C).

### 3.2. Calibration and Validation

The six-point cabozantinib standard calibration curve was expressed as y = 2.3644x + 0.0073 (coefficient of determination (R^2^) = 0.99999), where y is the peak height ratio and x is the concentration (in µg/mL). The calibration curves for cabozantinib were linear over the concentration range of 0.05–5 µg/mL. All intra- and interday coefficient of variation values were <3.26%. The intra- and interday accuracies for measuring cabozantinib concentration ranged from −4.35% to 0.79% and −1.05% to 0.98%, respectively (Table 1). The recovery rate was >96.04%. The LOQ and LOD for cabozantinib were 0.05 and 0.025 µg/mL, respectively.

### 3.3. Stability

Cabozantinib spiked into human plasma was stable for 6 h at 20 °C, 24 h at 4 °C, 1 month at −60 °C, and during three freeze–thaw cycles. The results of the stability experiments are shown in Table 2.

### 3.4. Survey of Concomitant Medications in Patients Who Received Cabozantinib Using Real-World Data

Cabozantinib was prescribed to 374 patients in the JMDC claims database between August 2021 and July 2022. The median age at baseline was 71 years (interquartile range: 64–77 years). The study population included 308 men (81.0%). Malignancies were mainly RCC (302 patients, 80.7% of the total) and HCC (72 patients, 19.3% of the total). The top 10 concomitant medications used are shown in Table 3. The most frequently prescribed concomitant drug was levothyroxine, followed by amlodipine.

### 3.5. Selectivity

Interference testing was conducted using five frequently concomitant drugs: levothyroxine, amlodipine, acetaminophen, furosemide, and esomeprazole. These five medications were obtained from Tokyo Chemical Industry Co., Ltd. (Tokyo, Japan). Working solutions of acetaminophen, amlodipine, cabozantinib, erlotinib (IS), esomeprazole, furosemide, and levothyroxine were prepared in methanol at concentrations of 0.5 mg/mL. Each concentration was selected to obtain similar peak heights in the chromatograms. No interfering peaks from the five medications were observed at the retention times of cabozantinib and the IS. The retention times for acetaminophen, amlodipine, esomeprazole, furosemide, and levothyroxine were 2.8, 2.9, 3.5, 3.2, and 2.9 min, respectively.

### 3.6. Clinical Application

**Figure 4:**
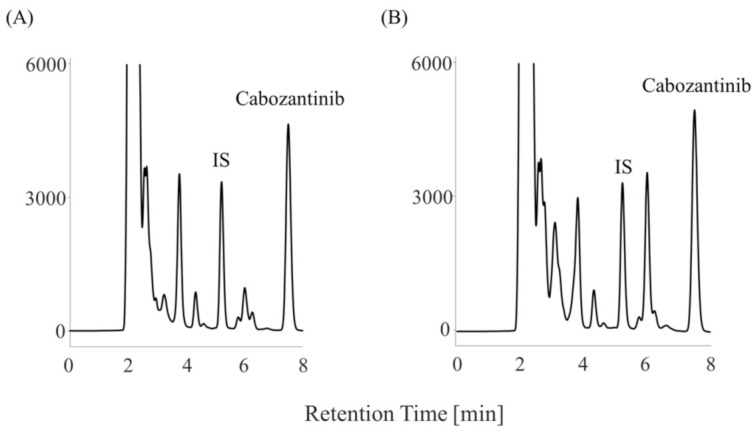
Chromatograms of a patient who received 20 mg of cabozantinib. (**A**) plasma sample and (**B**) serum sample.

The plasma concentration of cabozantinib was evaluated using samples obtained from a patient with RCC treated with 40 mg cabozantinib. The patient discontinued the 40 mg cabozantinib treatment because he developed hand–foot syndrome. Subsequently, the patient’s hand–foot syndrome improved, and she resumed treatment with 20 mg cabozantinib. Blood samples were collected before the administration of 20 mg cabozantinib on day 99. The plasma and serum concentrations of cabozantinib were 0.63 and 0.65 µg/mL, respectively (Figure 4). She was taking alendronate, atorvastatin, famotidine, losartan, nifedipine, prednisolone, sitagliptin, and voglibose as concomitant medications.

## 4. Discussion

Cabozantinib has been used to treat RCC and HCC. The plasma concentration of cabozantinib may vary occur according to factors such as age, weight, disease status, and drug interactions. TDM of cabozantinib in clinical practice can enhance treatment effectiveness and reduce toxicity. We developed a simple and accurate HPLC–UV method for measuring plasma concentrations of cabozantinib. Our methodology adhered to the US Food and Drug Administration guidelines [13] and showed a high level of accuracy, sensitivity, and a short sample preparation time.

Maruyama et al. [11] developed the first quantitative method for cabozantinib detection using HPLC–UV, reporting a quantitative concentration range of 0.02–4 µg/mL, which is lower than our quantitative concentration range. However, a therapeutic window between 0.5 and 1.5 µg/mL has been proposed for cabozantinib trough levels to ensure an acceptable balance between effectiveness and toxicity [14]. The plasma concentration of cabozantinib in Japanese patients receiving repeated doses of cabozantinib at steady state has been reported to be approximately 1.04–2.08 µg/mL [4]. We used our assay to measure the level of cabozantinib in the blood of a patient receiving 20 mg of cabozantinib; the plasma concentration was 0.63 μg/mL, which is comparable to the 0.53 μg/mL reported by Cerbone et al. [9], and showed clinical efficacy. A method for measuring cabozantinib concentrations in human plasma was developed using LC–MS/MS, with linearity over a concentration range of 0.025–5 µg/mL [10]. This method is superior to the previous method [11] in four respects. First, the time from sample preparation to measurement, including measurement time, is approximately 30 min less per sample. Second, this method has a small CV and high accuracy. The intraday and interday CV were 3.26% and 2.52%, respectively, with this method, compared to 5.2% and 6.8%, respectively, with the previous method. In addition, the intraday and interday accuracies were −4.35% to 0.79% and −1.05% to 0.98% [11], respectively, with this method, compared with 0.6% to 10.1% and 0.3% to 9.7%, respectively, with the previous method. Third, the initial investment is low and the procedure is simple because few instruments are used for sample preparation. Our method is simpler than the previous method and saves time because the supernatant liquid does not need to be dried under nitrogen vapor. Most hospitals do not have the equipment needed for nitrogen vaporization. In addition, the same organic solvent is used for deproteinization, but only 140 µL is required, compared with the 800 µL required with the previous method. Because less acetonitrile is used, the costs of purchase and disposal are lower. Fourth, this method uses fewer mobile phases. In the previous method, three mobile phases (acetic acid, ammonium acetate, and methanol) were used, but to simplify the method and prevent mistakes in the mobile phases, we used only two mobile phases (0.5% KH₂PO_4_ and acetonitrile).

Our method involves repeated injections of unfiltered supernatant solution directly into the HPLC system. Thus, it is a simple and inexpensive method that does not require the use of filters. One drawback, however, is that the long-term stability and performance of the column has not been evaluated. Therefore, we prefer to use filters. Finally, our method is rapid: sample preparation takes approximately 15 min, and the measurement takes 9 min. Our assay is also applicable to serum, and although we only studied one sample, we found that the plasma and serum concentrations of cabozantinib were similar.

The median daily dose of cabozantinib in the Japanese phase 2 trial (26 mg) [4] was lower than that in the international phase 3 trial (43 mg) [2]. The predicted steady-state average cabozantinib concentrations for starting dose levels of 60, 40, and 20 mg were 1.13, 0.75, and 0.38 µg/mL, respectively [6]. The method established in this study covers the range of 0.05–5 µg/mL (R^2^ = 0.99999); even when the dose is reduced to 20 mg, the trough concentration range is still within the LOD. Therefore, this method adequately covers the concentration range of cabozantinib. In addition, the required volume of plasma for cabozantinib detection is 50 µL with our method, compared with 100 µL using the LC–MS/MS method [10]. 

Pharmacokinetic–pharmacodynamic analysis of cabozantinib has shown that the response and safety are related to the level of exposure [6,15]; however, current evidence on the optimal target blood levels of cabozantinib is limited. Although TDM of cabozantinib is currently exploratory [16], the focus should be on establishing cabozantinib TDM as part of routine patient care. In our real-world study, the most frequently prescribed drug was levothyroxine, which is likely to be therapy for cabozantinib-induced hypothyroidism. Calcium blockers were the most commonly prescribed drug class for cabozantinib-induced hypertension, as in our patient. We also used real-world data to identify drugs often used concomitantly when prescribing cabozantinib, and the selectivity of these drugs for cabozantinib and the IS. The methods that we developed are suitable for use in clinical settings. It has been reported that 43–51.6% of patients with RCC are prescribed proton pump inhibitors (PPIs) during cabozantinib treatment [17,18]. A recent multicenter retrospective study reported that PPI use had a significant negative impact on progression-free and overall survival [18]. This has been attributed to a PPI-induced decrease in cabozantinib absorption and an associated decrease in blood drug levels [18]. PPIs were prescribed to 37% of patients who were prescribed cabozantinib in our real-world database. We plan to conduct a prospective study to examine the effects of PPIs on the pharmacokinetic and therapeutic outcomes of cabozantinib using our method to conduct TDM-based precision dosing of cabozantinib. The elimination half-life of cabozantinib is 99 h [14], and TDM of cabozantinib should be performed at least 15 days after the first cabozantinib administration. The acquisition of drug resistance is an important factor in the pharmacotherapy of RCC, contributing to poor prognosis and limiting the long-term response [19,20]. Therefore, our method of measuring plasma cabozantinib concentrations should be used to investigate the relationship between metabolic profiling [21] and cabozantinib blood concentrations, which affect prognosis and therapeutic efficacy.

This study had some limitations. Our method measured cabozantinib and IS in only one sample obtained from a patient receiving cabozantinib. Therefore, we used real-world data to identify frequently used concomitant medications and assess the interference between cabozantinib and the IS and frequently used concomitant medications. There is a need to confirm that cabozantinib and the IS can be accurately measured in the plasma of patients taking concomitant medications. Moreover, interference by metabolites of concomitant medications has not been investigated. Selectivity should be confirmed using clinical samples from multiple patients receiving cabozantinib.

## 5. Conclusions

We developed a simple, quick, and inexpensive HPLC–UV method for the quantification of cabozantinib in clinical settings. This assay could contribute to the personalization of medication in cancer patients undergoing treatment with cabozantinib in the clinical setting.

## Figures and Tables

**Table 1:** Intra- and interday accuracy and recovery.

	Intraday (*n* = 5)			Interday (*n* = 5)			
Theoretical Cabozantinib Concentration (µg/mL)	Detected (µg/mL) Mean ± SD	CV (%)	Accuracy (%)	Detected (µg/mL) Mean ± SD	CV (%)	Accuracy (%)	Recovery (%)
0.05	0.05 ± 0.002	3.26	0.79	0.05 ± 0.000	2.00	−1.05	96.04
0.1	0.10 ± 0.002	2.17	−1.36	0.10 ± 0.003	2.52	0.98	99.44
0.5	0.49 ± 0.010	1.97	−1.57	0.50 ± 0.005	1.01	0.94	103.00
1.0	0.97 ± 0.008	0.81	−3.39	1.00 ± 0.013	1.32	−0.19	101.49
2.5	2.42 ± 0.018	0.70	−3.17	2.50 ± 0.033	1.33	−0.15	103.70
5.0	4.78 ± 0.040	0.84	−4.35	4.98 ± 0.090	1.80	−0.38	104.52

SD, standard deviation; CV, coefficient of variation.

**Table 2:** Stability of the method for measuring cabozantinib.

	Stability Condition (%)			
Theoretical Cabozantinib Concentration (µg/mL)	Benchtop Mean ± SD	Short-Term 24 h Mean ± SD	Long-Term 1 month Mean ± SD	Freeze and Thaw Mean ± SD
0.05	92.77 ± 1.456	112.35 ± 3.009	101.74 ± 2.139	100.50 ± 3.412
1.0	100.81 ± 1.329	97.85 ± 2.013	97.53 ± 1.490	98.99 ± 0.516
5.0	97.51 ± 1.043	97.65 ± 1.648	95.28 ± 1.436	100.30 ± 2.056

**Table 3:** Top 10 concomitant medications in patients who received cabozantinib.

Medication	*n* = 374 (%)
Levothyroxine sodium	161 (43)
Amlodipine besilate	141 (38)
Ursodeoxycholic acid	88 (24)
Acetaminophen	87 (23)
Magnesium oxide	73 (20)
Vonoprazan fumarate	70 (19)
Esomeprazole magnesium	67 (18)
Furosemide	64 (17)
Febuxostat	60 (16)
Nifedipine	58 (16)

## Data Availability

The data presented in this study are available from the corresponding author upon reasonable request. Data are not available to the public due to the protection of personal information.
